# The Dignity of Medicine

**Published:** 1908-07-18

**Authors:** 


### The Hospital

A Journal of

The Medical Sciences and Hospital Administration.

New Series. No. 71, Vol. III. [No. 1143, Vol. XLIV.] SATURDAY, JULY 18, 1908

### The Dignity of Medicine.

It is no uncommon complaint that the dignity accorded to our profession falls short of that granted to other professions, notably the Bar. This is not so true at the present time as in the past, but there is some truth in it. In view of the notorious glamour of antiquity, the first explanation which rises to the mind is that modern medicine is a stripling relatively to the law. Although we may assume that the practice of healing is as old as the race we are forced to admit that medicine, regarded as a science, is barely three hundred years old. Granting that Hippocrates was all that is claimed for him by zealous historians—which is open to question when one reflects upon his precious humoral theory which obsessed the medical world for twenty centuries—there is little evidence of a science of medicine until the days of Harvey. The plain man may be excused for thinking that until this date the science of medicine, whatever its art may have been, was chiefly constituted by theories evolved haphazard from the inner consciousness of imaginative people, an extensive jargon of Latin and Greek, and a thick veneer of superstition and sorcery. The pompous mystery in which physicians wrapped their ignorance did no doubt impose upon a majority of people, but the occasional exposure of the pretence by penetrating individuals can hardly have advanced the dignity of its practitioners.

As regards the art of medicine in pre-Harveian days, we have an illuminating estimate of it from the pen of Montaigne. "As for me," he says, according to Florio's translation, "I honour Physitions, not according to the common-receiv'd rule, for necessitie sake, but rather for love I beare unto themselves; having seene some, and knowne diverse honest men amongst them, and worthy all love and esteeme. It is not them I blame, but their Arte; yet do I not greatly condemne them for seeking to profit by our foolishnesse, for most men do so and it is common to all worldlings. Diverse professions and many vocations, both more and less worthie than theirs, subsist and are grounded only upon publike abuses and popular errours. I send for them when I am sicke and they may conveniently be found; and love to be entertained by them, rewarding them as other men doe. I give them authority to enjoyne me to keepe myself warme, if I love it better so than otherwise. They may chuse, be it either leekes or lettuce, what my broth shall be made withall, and appoint me either white or claret to drink: and so of other things else, indifferent to my taste, humour, or custome." Certainly the Lord of Montaigne must have been a difficult patient for a practitioner with any sense of humour. In the third book, again, of his essays he continually recurs to the distresses he has endured, and the futility of medicine as regards relief from them. "Oh God," he cries, "that physicke would one day affoord me some good and perceptible hope, how earnestly would I exclaime *Tandem efficaci do manus scientiae*. The Arts that promise to keepe our body and mind in good health, promise much unto us; but therewith there is none performeth lesse what they promise. And in our dayes such as make profession of these Arts amongst us doe lesse than all others shew their effects. The most may be said of them is that they sell medicinable drugs; but that they are Physitions, no man can truly say it." And again, "They glut your eares with their Prognostications, and, surprising me heretofore when by my sicknesse I was brought very low and weake, they have injuriously handled me with their doctrines, positions, prescriptions, magistrall fopperies, and prosopopeyall gravity; sometimes threatening me with great paine and smart, and other times menacing me with neere and unavoydable death. All which did indeede move, stirre and touch me neere, but could not dismay, or remoove me from my place or resolutions. If my Judgement be thereby neither changed nor troubled, it was at least hindred."

Scientific medicine, then, is, as we have said, of comparatively recent date. Not so the law. Its genealogical tree is long enough and distinguished enough to satisfy the most exacting, and if some of its branches have in the far past disgraced the stock most scandalously, their presence but adds the same flavour of romance that a bar sinister adds to a shield of many quarterings. The law has always been associated in the nature of things with great doings and great personages. It has always commanded material power—power over acres, money-bags, and the liberty of individuals. It has thus throughout history lived within arm's reach of royalty, whether as slave or master, and in either case irradiated by the sun in its near neighbourhood. The high society in which it has moved, and the intimate connection between its just administration and the welfare of the public have both contributed to the endowment of the Bar with a dignity unusual to a learned profession; and the extent of the prizes which it offers, unmatched as they are both in emolument and titular distinction, has always attracted men whose intellect, ambition, and worth, though united in very varying proportions, have yet maintained the public dignity of their calling. Now democratic though we fancy ourselves to be, the popular status of a profession is still largely dictated by the height of the titular distinctions attainable by its members; and this, we have seen, is vastly in favour of the law. Moreover, the work of a doctor is essentially private, while the columns of the daily Press are a perpetual advertisement both of judges and of counsel. Until recent years the functions of our profession have been individual rather than national like the lawyers'; but the growing appreciation of the value of medicine, particularly in connection with the public health and the prevention of disease, justifies the anticipation that the future will see a redressing of the unequal balance. This reflection will not spoil our sleep, but no one who contemplates the triumphs of medicine and the national benefits resulting therefrom will grudge us the grumble noted in the opening lines of this article.

